# Maritime over the Horizon Sensor Integration: High Frequency Surface-Wave-Radar and Automatic Identification System Data Integration Algorithm

**DOI:** 10.3390/s18041147

**Published:** 2018-04-09

**Authors:** Dejan Nikolic, Nikola Stojkovic, Nikola Lekic

**Affiliations:** Vlatacom Institute, Bulevar Milutina Milankovića 5, Beograd 11070, Serbia; nikola.stojkovic@vlatacom.com (N.S.); nikola.lekic@vlatacom.com (N.L.)

**Keywords:** radar, HF radar, OTH radar, radar tracking, data integration, SAIS, LAIS, marine systems

## Abstract

To obtain the complete operational picture of the maritime situation in the Exclusive Economic Zone (EEZ) which lies over the horizon (OTH) requires the integration of data obtained from various sensors. These sensors include: high frequency surface-wave-radar (HFSWR), satellite automatic identification system (SAIS) and land automatic identification system (LAIS). The algorithm proposed in this paper utilizes radar tracks obtained from the network of HFSWRs, which are already processed by a multi-target tracking algorithm and associates SAIS and LAIS data to the corresponding radar tracks, thus forming an integrated data pair. During the integration process, all HFSWR targets in the vicinity of AIS data are evaluated and the one which has the highest matching factor is used for data association. On the other hand, if there is multiple AIS data in the vicinity of a single HFSWR track, the algorithm still makes only one data pair which consists of AIS and HFSWR data with the highest mutual matching factor. During the design and testing, special attention is given to the latency of AIS data, which could be very high in the EEZs of developing countries. The algorithm is designed, implemented and tested in a real working environment. The testing environment is located in the Gulf of Guinea and includes a network of HFSWRs consisting of two HFSWRs, several coastal sites with LAIS receivers and SAIS data provided by provider of SAIS data.

## 1. Introduction

Nowadays, it becomes clear that control of territorial waters is not enough to ensure secure flow of goods within the EEZ. The EEZ is a zone that stretches up to 200 nmi (approx. 370 km) from the territorial waters in the direction of the open sea. Within this zone, countries have exclusive rights such as the exploitation of biological and mineral resources from the sea [1]. Continually increasing organized crime and the growing threat of piracy make controlling the whole EEZ a must for every marine nation and not a privilege of only a few wealthy and economically developed countries. To the best of our knowledge, there are only two ways to achieve complete EEZ monitoring. The first approach utilizes optical and microwave sensors on platforms such as satellites and airplanes, thus avoiding range limitations of the sensors but introduces the platform’s limitations. The most limiting factor is interrupted data availability, since no airplane is able to stay in the air constantly and while satellites will be over the zone of interest for limited time only. The other approach uses a network of HFSWRs [2,3] to ensure constant surveillance well beyond horizon. Since the price of HFSWR radar network is significantly less than the combined cost of the aforementioned sensors and data is available constantly, it is clear why these radars slowly become the sensors of choice for maritime surveillance at OTH distances.

The data obtained via HFSWR must be processed before it can be integrated with AIS sources and the tracking algorithms are used for that purpose. There exist several types of tracking algorithms, which are approved for radars [4,5]. In order to have most accurate data, the complete radar data fusion process must include two essential algorithms: the tracking algorithm on a single radar level and the multi-radar multi-target data fusion algorithm. A radar network for over-the-horizon sea surveillance utilizes HF radars and introduces additional challenges to tracker design [2,3,6,7,8,9,10]. The algorithms which provide HFSWR data used here are described in [10,11].

When HFSWR data is fully processed and the unique target tracks are formed, the AIS [12,13] data can be assigned to the corresponding HFSWR tracks. Here, the following must be kept in mind:

- The latency of SAIS data could be very high and its accuracy is often questionable, so priority is given to HFSWR as source of target’s information.
- On the other hand, if there is a land AIS (LAIS) data source with good and easily verifiable accuracy and low latency, priority is given to the LAIS data.

The algorithm used for HFSWR-AIS data integration discussed in this paper strongly relies on algorithm described in [14]. While accuracy of the algorithm described in [14] is demonstrated on simulated data, the accuracy of algorithm described here is demonstrated with data obtained from sensors working in a real operational environment, the Gulf of Guinea. It is worth noting that algorithm described in this paper already passed the final stage of operational testing and it is used every day.

The rest of the paper is organized as follows, in Section 2 we are describing the operational environment. In Section 3 we provide a description of the integration algorithm. The field results are presented in Section 4 and statistical analyses in Section 5. Conclusions are drawn in Section 6 and as attachment to this paper two appendices are added.

## 2. Operational Environment and Sensor Network

The environment where the whole system is deployed is the Gulf of Guinea. From our point of view, this environment is one of the most challenging in the world for the task we are targeting because of natural and man-made challenges.

• Natural challenges:

The Gulf of Guinea is located in the equatorial area where natural noise levels are greater than in any other part of the world [15,16]. Also, very frequent gales occur on a nearly daily level in this region. Radar range is decreased due to the noise levels and the gales make detecting weak echoes difficult during gales, since surface wave propagation over a rough sea introduces a new attenuation factor into the radar equation. 

The aforementioned natural conditions make detection of vessels on the open sea a challenging task. The tracking algorithm at the radar level, described in [10] provides a solution for maintaining an unstable track, while multi-radar multi-target algorithm described in [11] forms unique radar tracks from all sources. More about natural challenges may be found in Appendix A of this paper.

• Man-made challenges:

Most of the challenges are a direct consequence of the lack of a strong regulatory body which defines the rules in the maritime arena. This leads to situations where AIS devices are very often turned off and their technical correctness may be questionable. Moreover, turning an AIS device off is often used as preventive measure which reduces chances of piracy attacks. Furthermore, transmitting another vessel’s AIS data is a practice used during smuggling. At the end, SAIS data latency can be very high, practically sometimes it can be measured in hours. 

On the other hand, underdeveloped communication networks make data delivery quite hard. Since developing a full communication network for large areas only to provide consistent data for maritime surveillance is far from cost effective, data delivery is achieved through satellite communications. This introduces data delivery problems during bad weather conditions due to the increasing number of lost packages. 

**Figure 1:**
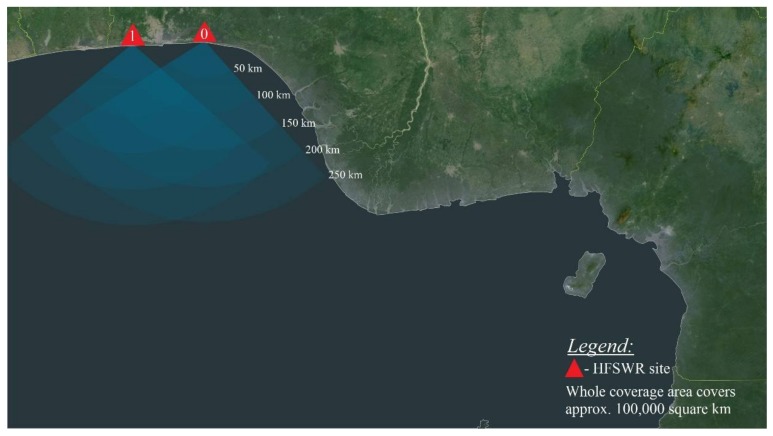
High frequency surface-wave-radar (HFSWR) network coverage area.

All the aforementioned challenges make forming of unique operational picture at OTH distances in a Gulf of Guinea quite a difficult task. In order to provide the needed data, the following sensor network is formed:

- Two HFSWRs with a nominal range of 80 nautical miles (approx. 150 km) for a Bonn express class of vessel [17] during night-time and sea states [18] up to 3. For larger vessels and during the day—time range can extend even beyond 125 nautical miles (approx. 230 km). Regardless of the time of day and vessel size, angle coverage is set to 120 degrees. More about used HFSWRs can be found in [19], while the HFSWR network coverage area over the Western part of Gulf of Guinea and it is shown in Figure 1,
- Six coastal sites equipped with AIS receivers and
- Data provided from SAIS provider, Orbcomm [20].

In order to effectively combine data, all data is fed to the main data server located in a command and control center. The server performs three operations: tracking on single radar level, fusion of data from multiple HFSWRs and integration of SAIS and land AIS data with data provided from the fusion software layer. Although forming a single radar track in the main data server instead at the radar site might look less than optimal, it is the best solution in the given environment. Firstly, transferring plots from the radar site to the main server consumes less bandwidth than transferring the whole tracks. Secondly, the loss of a package of plots impacts the whole system far less since all tracking is done at the main data server.

At the end of this paper, in Appendix B, sensor accuracy is briefly discussed and demonstrated on one example.

## 3. Description of Data Integration Algorithm

This algorithm’s inputs are Multi-Radar Fusion Track (MRFT) points and AIS data. MRFT points (MRFTP) are the product of a multi-radar multi-target tracking algorithm and represent current positions of vessels observed by the network of HFSWRs. Every MRFT is defined by its identification number—ID number, while AIS data have their unique identifiers—Maritime Mobile Service Identity (MMSI) [21]. Output of the proposed algorithm is a set of unique data pairs (AIS MMSI, MRFT ID number) that are considered to originate from the very same vessel. Since, matching of the aforementioned pairs is never 100% accurate, for every data pair, the probability of matching is calculated. Based on this probability the final decision is made.

The proposed algorithm is triggered by reception of a new MRFTP list and so-far collected AIS messages. MRFTP dataflow is periodic with regular repetition cycles (33 s). On the other hand, AIS message dataflow is chaotic, with great uncertainty in timing of message delivery, especially in the case of Satellite AIS transmissions, where message deliveries could have delays measured in hours. Such behavior implies that, in order to perform matching of AIS data with MFRTs, 8 h of deep history records for every MRFT and AIS must be kept.

**Figure 2:**
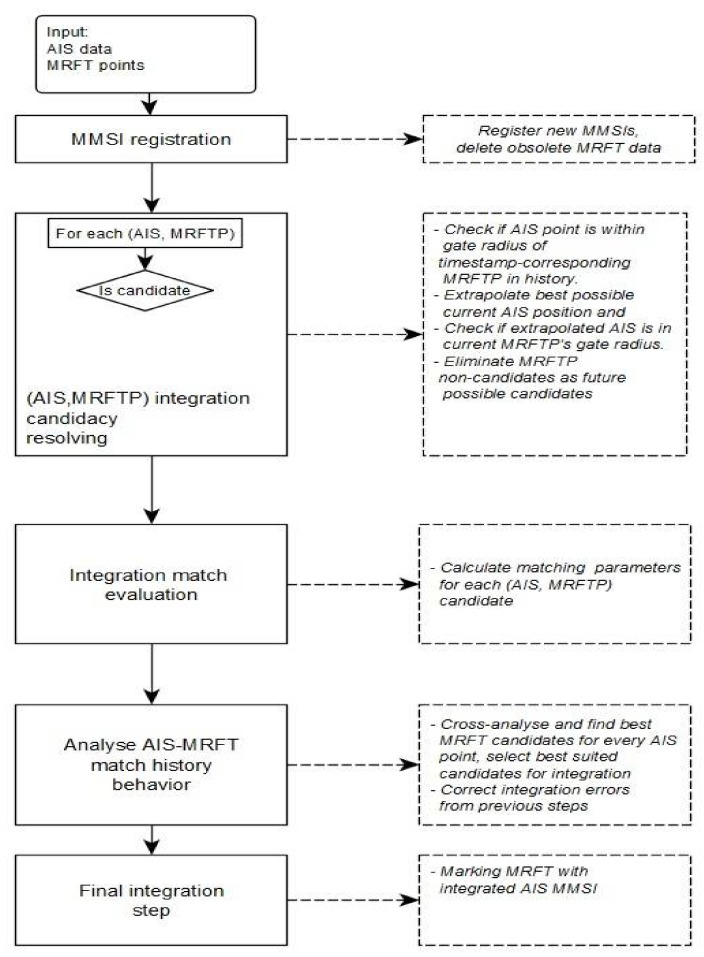
Steps of automatic identification system-multi-radar fusion track (AIS-MRFT) integration algorithm (taken from [14]). In the first step, the algorithm is conducting the following operations.

Steps of the proposed algorithm are shown in Figure 2.

- creating new entries for MMSIs which are reported for the first time,
- refreshing data for already existing AIS data-MRFT pairs, if it is possible and
- cleaning up old and unneeded data.

**Figure 3:**
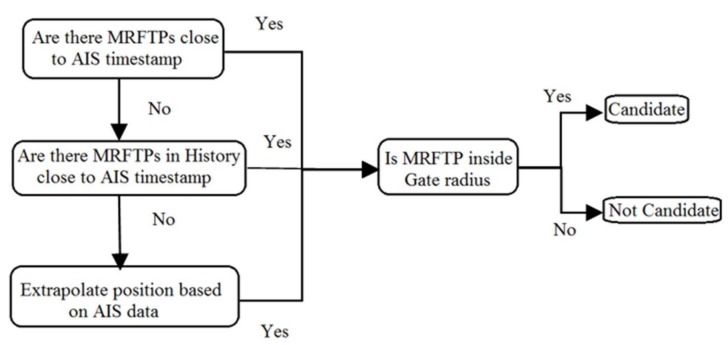
Step 2 of AIS-MRFT integration algorithm—Search for Candidates.

During the second step the algorithm is searching for a set of suitable MRFT integration candidates for each AIS data source. “This search” is shown in Figure 3.

First, the algorithm checks all currently active MRFTPs in order to find the ones with a timestamp close to that of the AIS data. If this cannot be achieved, the algorithm tries to load MRFTs from its history in order to find those MRFTPs close to the AIS timestamp. If this, cannot be achieved, the AIS position is extrapolated. Extrapolation is done with the data provided by AIS under the assumption that the vessel did not change its course and speed. In this way, the AIS position is translated to the current time.

Next, the gate radius needs to be checked. Those MRFTs which pass both checks (timestamp and gate radius) are considered to be candidates for the integration. Please note that it is possible that one AIS data has multiple MRFT integration candidates and vice versa. All those MRFTs which did not pass previous checks are considered unsuitable for integration with observed AIS data. If MRFT is considered unsuitable for integration N consecutive times, observed MRFTs shall never again be considered for integration with the observed AIS track. In this way, the algorithm cuts unnecessary operations and this speeds up future processing. 

In the third step, for each candidate pair, the matching factor is calculated. The matching factor (*M*) represents the likelihood that the observed pair of data originates from the same vessel. It consists of speed and course matching factors, Equation (1).

(1) M=Mv×Mc

where *Mv* and *Mc* represent speed and course matching coefficients for the candidate pair. The speed matching coefficient is defined with Equations (2) and (3), while the course matching coefficient for the candidate pair is defined with Equations (4) and (5).

(2) $$v_{diff}=\left|v_{AIS}-v_{MRFT}\right|$$

(3) $$M_{v}=1-\frac{v_{diff}}{greater\_of(v_{AIS},v_{MRFT})}$$

where *v_AIS_* and *v_MRFT_* represent the vessel’s speed reported by AIS and the HFSWR network respectively, while *v_diff_* is their difference.

(4) $$C_{diff}=\left|C_{AIS}-C_{MRFT}\right|$$

(5a) $$M_{c}={\left(1-\frac{C_{diff}}{180}\right)}^{2},\;\mathrm{for}\;0\;\leq \;C_{diff}\;\leq \;180$$

(5b) $$M_{c}={\left(1-\frac{C_{diff}}{360}\right)}^{2},\;\mathrm{for}\;180\;\leq \;C_{diff}\;\leq \;360$$

If *C_AIS_* and *C_MRFT_* represent the vessel’s course reported by AIS and HFSWR network respectively then *C_diff_* is their difference. *M_c_* represents the course matching coefficient for the candidate pair. Due to the nature of the course notation, two equations are a must for the definition of the course matching coefficient. 

At the end of this step, for every AIS track MFRT candidates are sorted by their matching factor value.

During the fourth step, mutual integration affinities are cross-analyzed. Parameters which are taken into account are:

- Current matching factor,
- Average value of matching factor during previous N integration periods and
- Number of candidates.

At the end of this analysis, the old integration links may be broken or confirmed, while some new integration links may be created. Finally, the best possible integration pairs of data are chosen and processed further, while uniqueness of integration pairs (AIS MMSI, MRFT Id) is preserved. It is important to note that the integration link will not be created if the matching factor is less than 51%. In other words, the threshold level for the integration link creation or confirmation is 51%.

At the final stage, all integrated MRFTs are marked with their corresponding MMSI. It is important to note that all MRFTs which cannot be integrated with any AIS data will be forwarded as they are. The same stands true for all AIS data which cannot be integrated with any MRFTs.

## 4. Discussion

Before beginning the discussion regarding the field results, a few things need to be noted:

1. The graphical environment presented here is used just for the data visualization of the described integration process, not as the command and control software’s GUI.
2. Since tests took over a year, the visualization environment underwent some changes during this time, hence there are some minor graphical differences between the figures presented in this chapter. Anyhow, the yellow vessel markers always represent MRFTPs, while white vessel markers always represent AIS data. Hexagonal encirclement around the vessel marker means that that marker is selected by an operator and details about the vessel are shown in a separate window. Trace colors behind vessel markers are random and thus don’t have any particular significance.

In order to demonstrate the algorithm’s capabilities in a real working environment, several representative situations will be shown and described in this chapter. These situations are:

1. A so called “clear situation,” that is, a single stable MRFT and corresponding single AIS dataflow with low latency. Here two cases will be examined:
  - Vessel is sailing in a straight line
  - Vessel is maneuvering
2. MRFT data inaccuracy
3. SAIS and/or LAIS data latency
4. Multiple MRFTs within single AIS data gating radius
5. Multiple AIS data with in single MRFT gating radius
6. AIS data absence, that is, only MRFT data and
7. MRFT data absence, that is, only AIS tracking

**Figure 4:**
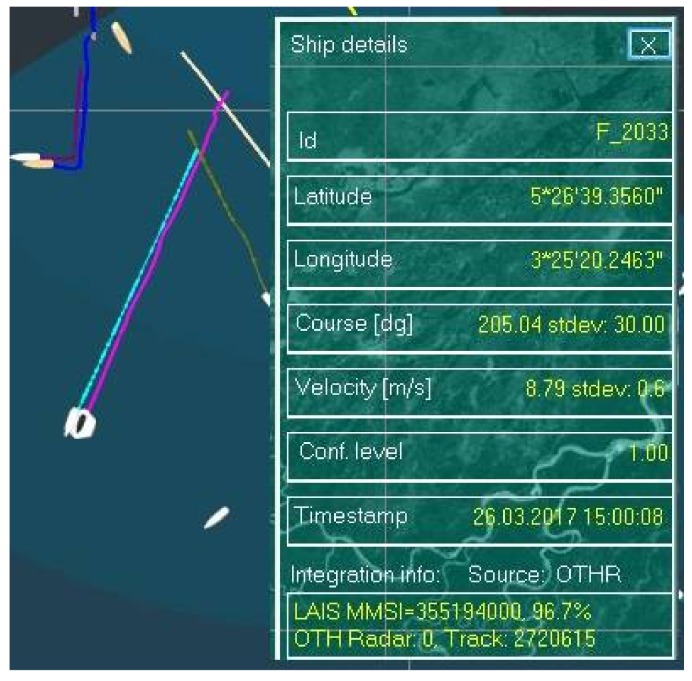
Resolving a “clear situation”—vessel is sailing in a straight line (light purple trace MRTF, light blue trace AIS).

Firstly, a so called “clear situation” will be discussed. A stable MRFT and low AIS data latency (latency is measured in minutes) are main characteristics of clear situations. Since there is no need to load data form history and/or extrapolate AIS data, the whole integration process is simplified. In Figure 4 a clear situation with a vessel sailing in a straight line is presented. The MRFT labelled with Id F_2033, originating from the radar 0, radar track ID No. 2720615, is a candidate for integration with its AIS source defined with MMSI 355194000 in its vicinity. Since the matching coefficient is very high (96.7%) both data points most likely originate from the same vessel and they will be fused into one track in further processing. Please note that AIS data latency in this case is barely a few minutes which makes integration process quite easy, since the tracked vessel moved only a few hundred meters. This movement is insignificant since the MRFT’s gate radius is around 1 km (current MRFTP and the received AIS data is still within the same gate). 

**Figure 5:**
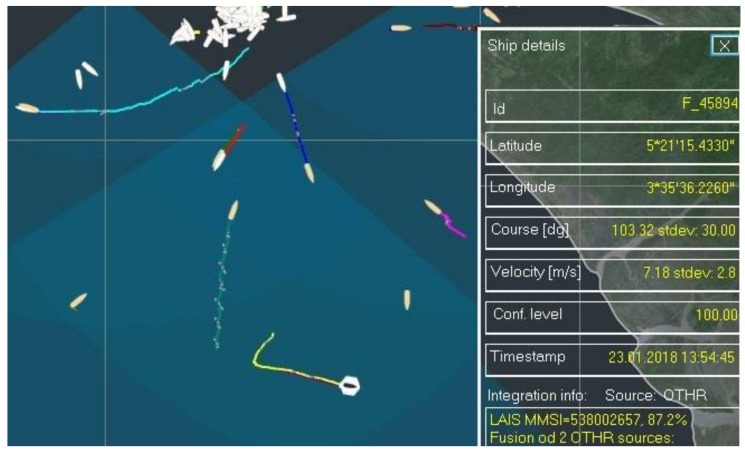
Resolving a “clear situation”—maneuvering target (yellow trace: MRFT data, dark red trace: AIS data).

Next, a maneuvering target will be considered and thus the implications of maneuvering on the integration process will be discussed. During maneuvering, the vessel changes its course and speed, the basic parameters used are matching factor calculations, which implies that matching factor value will drop accordingly. Here even a minute of AIS data latency stands out and has clearly visible implications on the integration process. In other words, due to the latency of the data, course and speed that are measured by HFSWR and reported by AIS can significantly differ. This leads to situations where the matching factor will drop temporarily, since the newly calculated matching factor will be quite low which will decrease the average value significantly. After the vessel stabilizes its course the matching factor will rise again. As an example, we may analyze the situation presented in Figure 5. The MRFT with ID number F_45894 abruptly changed its course by 90 degrees which lead to a fall of the matching factor. After the vessel stabilized its course, its speed and course that was reported by AIS and MRFT came to similar values and the matching factor came to 87.2%. It is important to note that during this temporary fall of the matching factor, the integration link was not broken and uniqueness of integration of the pair has been preserved. 

Now, a very interesting situation will be examined. This particular situation very nicely demonstrates the capabilities of the algorithm, since it includes the following challenges:

1. MRFT data inaccuracy,
2. Multiple MRFTs within single AIS data gating radius,
3. High SAIS data latency and
4. AIS data absence, that is, only MRFT data

**Figure 6:**
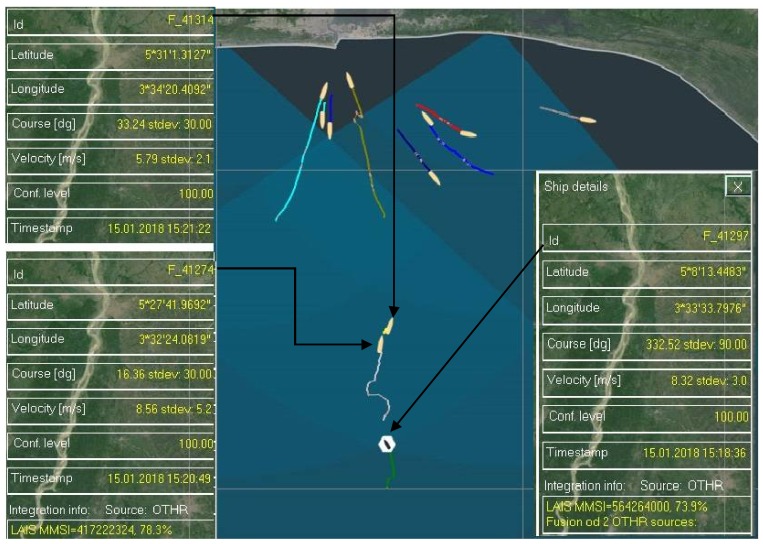
Resolving a complex situation—only MRFT data presented.

Firstly, in Figure 6 only MRFTs will be presented.

From Figure 6 it could be noticed that there are two stable MRFTs, labeled with F_41297 and F_41274. Both MRFTs are integrated with their corresponding AIS data designated by 564264000 and 417222324, respectively. Then, another vessel was detected by HFSWRs and Id number F_41314 was assigned to it. 

**Figure 7:**
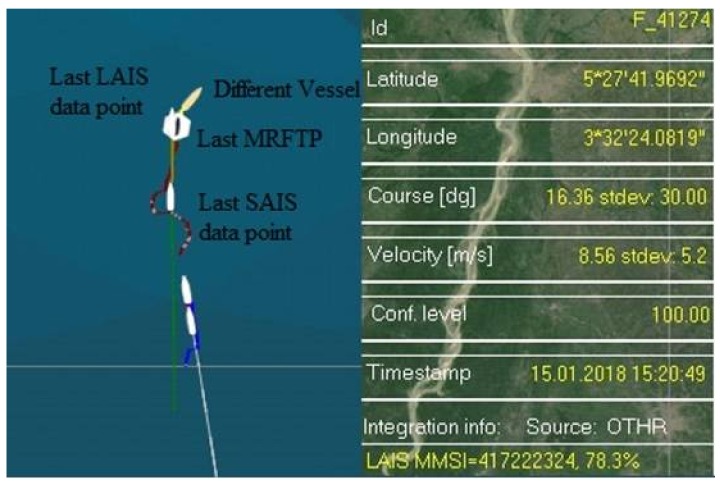
Resolving a complex situation—MRFT F_41274 (dark red trace: MRFT data, green trace: AIS data).

MRFT labeled F_41274 will be examined more thoroughly. Based entirely on the HFSWR data it could be concluded that this vessel was conducting nearly a zig-zag manoeuver (Figure 6). It is highly unlikely that this was the case, so available AIS data will be examined as well (Figure 7).

From Figure 7 it could be easily seen that there are long and stable trace produced by LAIS data. In this case there is no LAIS data latency, so the data provided by the LAIS feed may be considered highly accurate. On the other hand, SAIS data arrives with significant latency and may be discarded during further processing, since there is a stable LAIS data feed. Although stable, MRFT shows high inaccuracy and may not be considered as accurate representation of the vessel’s trajectory. So, the vessel was sailing in a straight line and no maneuvers were conducted by the vessel. This is a clear case of MRFT data inaccuracy, as well as SAIS data latency. Moreover, detection of a new target by HFSWRs in a same AIS data gating radius put the integration algorithm to another test. Precisely, the algorithm needed to resolve which MRFT is the best candidate for the AIS data integration. This is done in the manner described in Section 3 of this paper and the result is presented in the Figure 6 and Figure 7. Finally, when decisions about data integration are made, the question arises about what to do with the MRFTs which cannot be integrated with any available AIS data. As it is described in Section 3, the algorithm will forward this MRFT as it is and final result is shown in Figure 6 and Figure 7.

**Figure 8:**
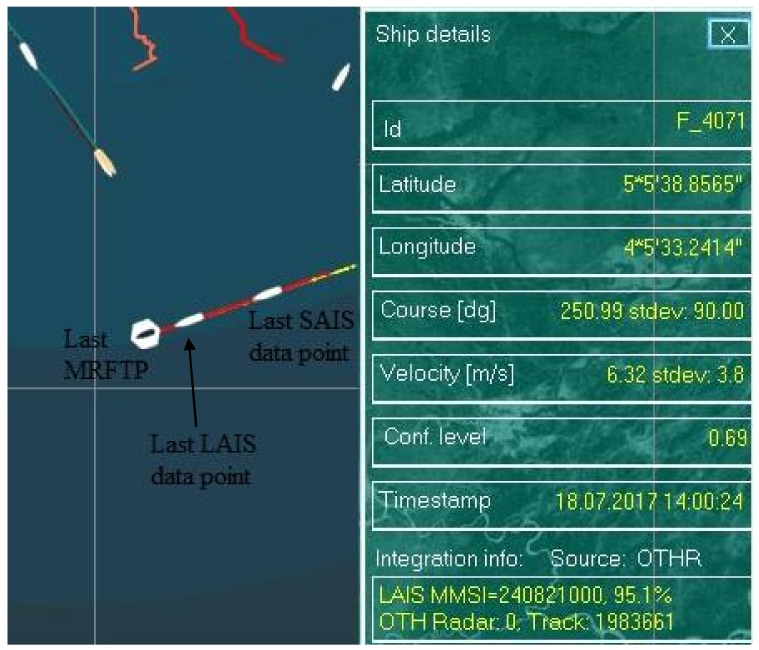
Land automatic identification system (LAIS) and satellite automatic identification system (SAIS) data latency (red trace: MRFT data, yellow trace: AIS data).

Next, we will examine a situation where both LAIS and SAIS have significant data latency (Figure 8).

From Figure 8 it can be seen that the LAIS data has significant latency in comparison to the MRFTP, while SAIS data latency is even greater. In this case it is obvious that algorithm needs to make a decision based on a prior knowledge and data extrapolation. From Figure 8 it can be observed that there are two traces, the red one representing MRFT (labelled F_4071) and the yellow one which represents the track formed by the AIS data (MMSI: 240821000). It is clear that integration link between F_4071 and MMSI 240821000 already existed and this represents a prior knowledge in the current timestamp Based on this, AIS data is extrapolated and since there are no other MRFTs in the vicinity of the extrapolated AIS data, the algorithm decided that integration link shall be confirmed with a probability of 95.1%. In this way 3 different data sources about a vessel are merged into a single data feed despite their timestamp difference. 

The final scenario shows the proposed algorithm responses when multiple AIS data is within a single MRFT gating radius and when there is an absence of MRFT data, that is, only AIS tracking is available.

**Figure 9:**
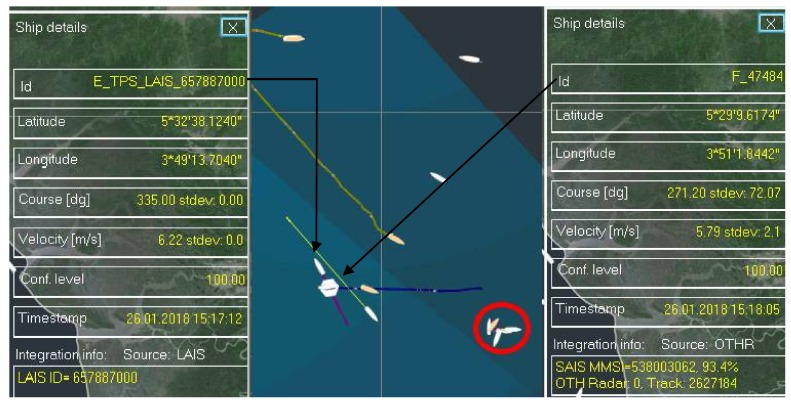
One MRFT and multiple AIS.

In the situations where there are multiple AIS sources within single a MRFTP gate radius (Figure 9) the algorithm will try to integrate AIS data which has the best matching factor with the MRFT. Although this looks like the right approach, it is not always the case. The situation encircled in red in Figure 9 is actually an oil platform with vessels around it. It is obvious that HFSWR detects all of them as a single target (since all the objects are inside one resolution cell) thus making an integration of a single AIS data feed to the mentioned MRFT meaningless. On the other hand, the complete different situation occurs when there is a stable AIS-MRFT data pair and unintegrated AIS data appears in its vicinity. In this case the algorithm will confirm an existing link in a process similar to the process when there are multiple MRFTs and only one AIS. When all the integration decisions are made, all unintegrated AIS data will be forwarded as they are (Figure 9 left data window).

## 5. Statistical Analysis

In this chapter, the statistical analysis of a number of targets received from the sensors in the network and number of targets delivered to the C2 system is presented. In other words, this analysis shows the percentage of duplicate targets eliminated by the presented algorithm.

Before analysis starts, a few things need to be pointed out:

- The first HFSWR was commissioned in mid-January 2017, the second HFSWR in mid-September 2017. All Coastal sites are added to the network as they were commissioned, starting with 4 in mid-January and adding 2 more in mid-September. Due to this total number of targets will rise during the time.
- Power supply issues are a common fact in Gulf of Guinea, not isolated incidents. Despite the fact that all sites are equipped with UPSs which can power the equipment for approximately 24 h, some sites are located in remote areas and cannot be reached within 24 h. This leads to the situations where a significant drop of the number of detected targets from one site are present, simply because the site was down.
- A similar situation occurs when there is a major problem with the satellite links. The problem mostly occurs when storms are raging in a certain area and block satellite communications from sites located in that area. This also causes a significant drop of targets as long as the link is down.

**Figure 10:**
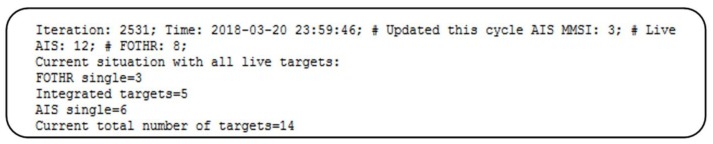
One iteration log file.

In Figure 10 one log file from our system is presented.

Explanation of the log file fields:

- Iteration—number of iteration process during single day
- Time—time and date when the log file was created
- Update this cycle AIS MMSI—number of AIS massages received during this iteration process. Basically, number of AIS massages received in last 33 s
- Live AIS—number of MMSIs which received at least one update in last one hour
- FOTHR—number of active MRTFPs
- FOTHR single—number of MRFTPs which cannot be integrated with any live AIS
- Integrated targets—number of integrated targets
- AIS single—number of MMSIs which cannot be integrated with any live MRTFP
- Current number of total targets—number of targets delivered to C2 system

Based on data displayed in Figure 10 the following can be concluded:

1. Total number of targets received from all sensors is 20,
2. Total number of targets delivered to C2 system is 14,
3. Number of eliminated targets is 6.

This means that 30% of all targets were duplicated and could cause false alarm triggers in C2 system.

**Figure 11:**
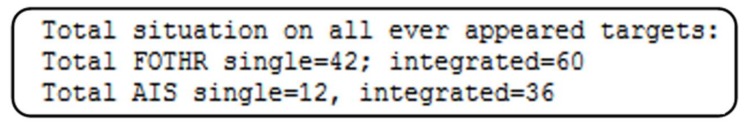
Day log file.

The log files were collected during the day in order to present the statistics for that day. The day log file is presented in Figure 11.

So for one day, the HFSWR network detected 102 targets, while 48 targets were collected through AIS, meaning that in total, 150 targets were received from the sensor network during that day. 42 HFSWR targets couldn’t be integrated with any AIS data and 12 AIS data did not have the corresponding HFSWR tracks, while for 60 HFSWR tracks 36 AIS data sources were found. Overall, 114 targets were delivered to the C2 system; 42 HWSWR, 12 AIS and 60 integrated. This means that 36 targets were eliminated during that day, or 24% of all received targets were duplicated. Please note, in this particular case there were more integrated HFSWR tracks than AIS data. Situations vary from day to day. 

**Figure 12:**
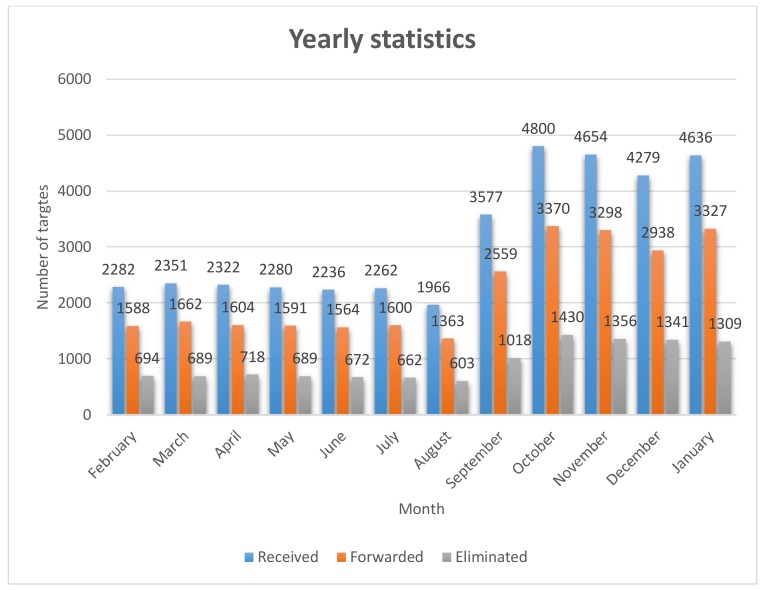
Yearly statistics.

Day log files are collected on monthly basis starting from the 1 February 2017 and ending with the 31 January 2018 in order to create a statistical analysis presented in Figure 12. 

Form Figure 12 it can be seen that around 650 duplicate targets were eliminated every month from February 2017 up to September 2017. A significant drop of targets can be noted during August, this is due to power issues with the HFSWR (at that time only one HFSWR in the network was functioning). During September, the second HFSWR was commissioned and 2 more coastal sites were added to the network, so the number of targets raised significantly. The drop which can be noted during December was due to the Holiday season. Overall, it can be concluded that the presented algorithm eliminates approximately 30% of received data, since they are duplicate. This significantly reduces false alarms.

## 6. Conclusions

In this paper we presented, described and tested an algorithm for the EEZ Monitoring at OTH distances based on HFSWR and AIS data integration. The testing environment is located in the Gulf of Guinea and includes a network of HFSWRs consisting of two HFSWRs, several coastal sites with LAIS receivers and SAIS data provided by the provider of SAIS data. The proposed algorithm is knowledge based and uses prior tactical knowledge in order solve situations in the field. After comprehensive tests, which took over a year, it can be said that the proposed algorithm shows high reliability in spite of its simplicity. This approach yields more accurate tracking, prevents the creation of duplicate targets and forms a complete operational picture at OTH distances with minimal computational costs. For future work, we intend to expand the algorithm’s capabilities and include coastal radars (S, X and Ku band radars), thus forming a complete operational picture from the coastline up to the end of EEZ.

## Figures and Tables

**Figure A1:**
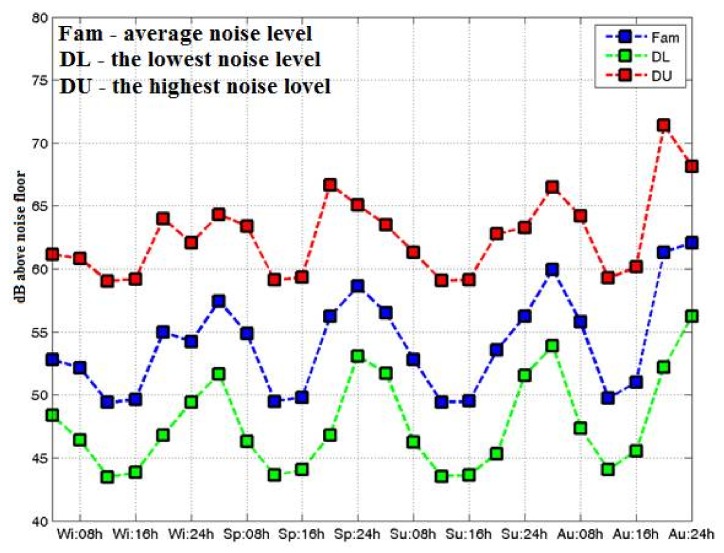
HF noise level in Gulf of Guinea.

**Figure A2:**
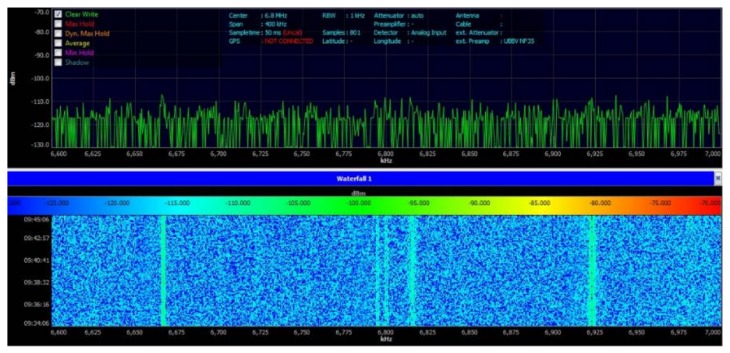
Measured noise level—Gulf of Guinea, 24 November 2016.

**Figure A3:**
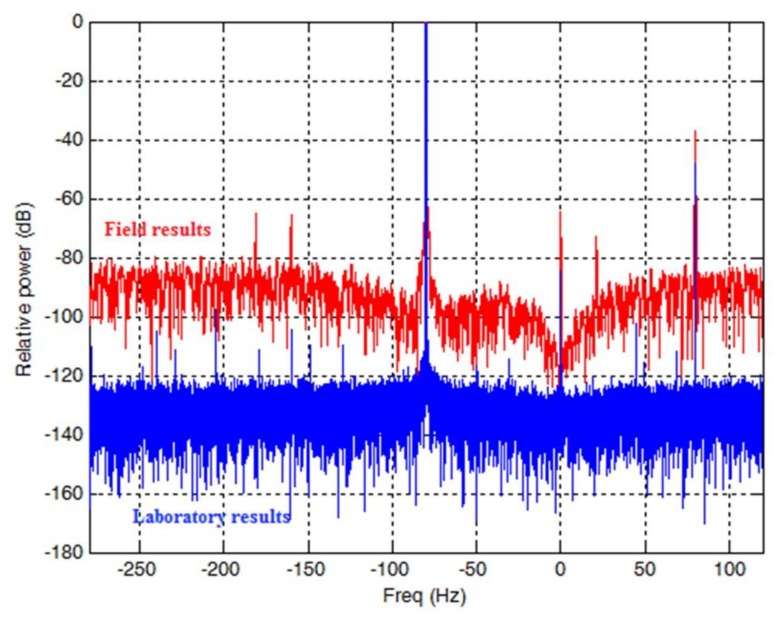
SNR after signal processing (blue trace laboratory test, red trace field test).

**Figure A4:**
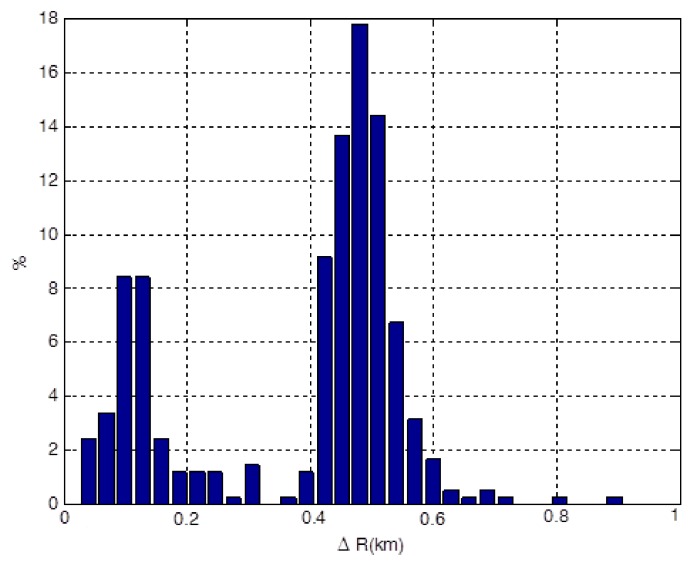
Range accuracy.

**Figure A5:**
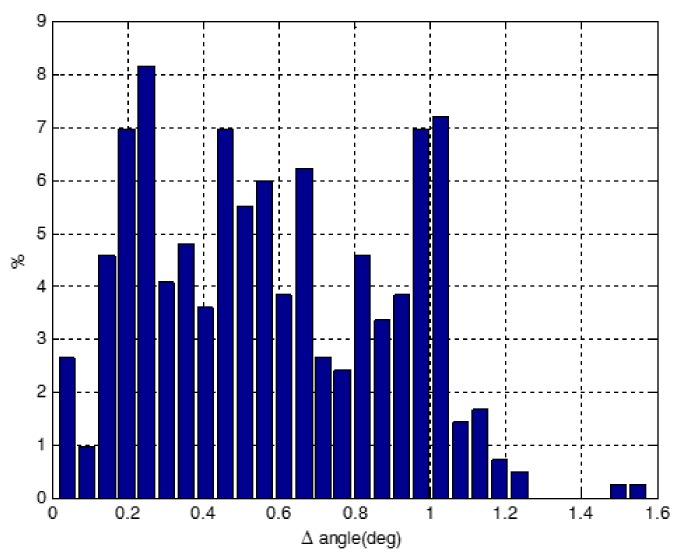
Angular accuracy.

**Figure A6:**
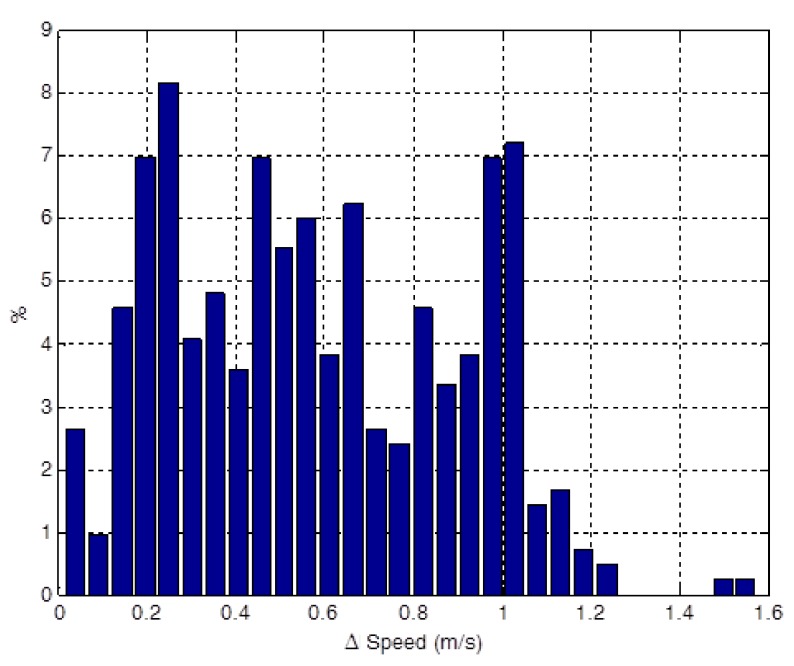
Speed accuracy.

## Appendix A. Noise in HF Band: Gulf of Guinea

The first document that any HF system designer should consider is the ITU-R Recommendation P.372-11, issued September 2013. Here, charts and graphs describing HF noise levels all over the world can be found. (Alternatively, the very same can be achieved with software package GHNoise (http://www.greg-hand.com/noise/)). It is important to note that noise in the HF band depends on the season and time of a day.

Based on this data, noise levels on a given location can be estimated (see Figure A1).

Figure A1 Clarification:

- Wi—Winter, Sp—Spring, Su—Summer and Au—Autumn
- 08 h, 16 h and 24 h represent time of a day,
- Y axis values represent noise power above noise floor (−174 dBm//Hz)
- Values are plotted for 6.8 MHz frequency

According to Figure A1 average noise level varies between −125 and −112 dBm/Hz. Measurements from the field (Figure A2) confirm the validity of the data presented in Figure A1. These measurements are taken using a monopole antenna centered at 6.8 MHz (VSWR less than 1.1) and a low loss cables.

Taking into account that the HFSWR receiver bandwidth is 1 MHz, the average noise level the radar needs to handle varies between −65 and −52 dBm/MHz.

In order to demonstrate higher noise level effects on the radar we need to compare SNR levels after signal processing in the laboratory and in the field.

- Setup for the laboratory measurements consists of a radar transmitter with attenuators (needed to simulate signal path) and a radar receiver with signal processing.
- Setup for the field test consists of an operating radar and the surrounding environment.

The test signal was run through the both setups and obtained results are shown Figure A3.

From Figure A3 it can be seen that the SNR level measured in the laboratory is 120 dB, while the SNR level in the field is barley 80 dB. It can be seen that the additional noise reduces the system’s dynamics by nearly 40 dB, which directly reduces the radar range.

## Appendix B. Sensor Accuracy

Here, sensor accuracy will be discussed.

For this analyses, track is taken into account only if both sources (AIS and HFSWRs) provide stable data. AIS data is taken as more accurate one and HFSWR data is compared to it. Methodology is following:

1. When both HFSWR and AIS are stable there is more data points originating from AIS than MRFTPs. So, only the AIS data point which is the closest to corresponding observed MFRTP will be taken into consideration.
2. Since AIS data point is considered more accurate, range error is calculated for MRFTP. Same is true for angle and speed errors.
3. For this analyses Hamilton class cutter (https://en.wikipedia.org/wiki/Hamilton-class_cutter) sailing approximately 40 to 60 nautical miles of shore is observed during 4 h period.
4. Data obtained during observation (417 data MRFTP-AIS pairs) are presented below.

The first range accuracy will be discussed.

Majority of tracks provided by HFSWR network have insignificant range error, since majority of range errors are lesser than 0.6 km (see Figure A4). This can be expected since HFSWR declared range error is less than 0.5 km. Next, angular error will be presented.

From Figure A5 it can be noted that angular error is mostly less than 1.2 degrees, which is in accordance with declared values (up to ±1.2°). At the end speed error will be discussed.

Since precision of vessel’s radial speed measurement is declared to ± 1 m/s, data present in Figure A6 confirm declared values.
